# 

**Published:** 1881-04

**Authors:** 


### ASSOCIATION.

The seventeenth annual meeting of the Illinois State Dental Society will be held at Rock Island, commencing Tuesday, May 10th, 1881 and continuing four days.

The following subjects are on the programme, for essays and discussions. Each subject is to be presented by a person assigned to that duty, after which all, who desire, will have an opportunity to take part in the discussion.

SUBJECTS AND ESSAYISTS.

1. Treatment of teeth containing dead and dying Pulps; also the treatment of Aveolar Abscess.
   *Dr. H. H. Townsend*, Pontiac.
2. Mechanical Dentistry; its past, present and future.
   *Dr. L. P. Haskell*, Chicago.
3. The development of the Enamel.
   *Dr. M. S. Dean*, Chicago.
4. Fractures of the Inferior Maxilla.
   *Dr. T. L. Gilmer*, Quincy.
5. Dental Hygiene. *Dr. W. P. Richards*, Elgin.
6. The Chemistry of Mercury and its constitutional effects, resulting from the use of Amalgam Fillings and Red Rubber Plates. *Dr. E. S. Talbot*, Chicago.
7. What must be the preparation for the successful practice of Dentistry in the future.
   *Dr. C. A. Kitchen*, Rockford.
8. Operative Dentistry. *Dr. G. S. Miles*, Jerseyville.
9. Report on the Saliva. *Dr. C. W. Spalding*, St. Louis.
10. Characteristics of Saliva in Syphilitic patients.
    *Dr. A. W. Harlan*, Chicago.

The indications are, that this will be an exceedingly interesting and profitable meeting. There will doubtless be a large number in attendance.

The following features have been arranged and will be carried out:

The clinics will be conducted by six operators each clinic day, thereby enabling every one present to witness a great variety of methods, and operations.

Dr. Rohland will demonstrate the use of the Electro-Magnetic Mallet. Dr. Swain will adjust artificial crowns upon natural roots, demonstrating several methods. Dr. Call will demonstrate his method of adjusting gold crowns. Dr. Greene will demonstrate the use of soft or non-cohesive gold. There will be an exhibition of the most improved methods of constructing artificial teeth, with a complete outfit for a model dental laboratory, by Dr. Haskell. Dr. W. N. Morrison of St. Louis, will extract, fill and replant a tooth; also, if a case can be provided, extract, fill and transplant one or more teeth.

A few prominent operators will select sets of instruments such as they use, and place them on exhibition. There will be a large collection of anatomical and pathological preparations exhibited, as well as a model Dental Library.

### INCIDENTS OF OFFICE PRACTICE.

An opportunity will be extended to those who have had cases in practice of unusual interest, to briefly present them to the society on Wednesday evening. Gentlemen desiring to present such cases are requested to previously meet the executive committee.

The hotels will give greatly reduced rates to those attending the meeting.

It is difficult to concieve more attractive inducements than are here offered. The subjects are eminently practical, and will be handled by practical and thoroughly competent men. Interesting clinical demonstrations are to constitute a promanent feature, the importance of this is shown, when it is remembered that many learn far more readily through the eyes than through the ears. A cordial invitation is extended to the prefession, not only in, but outside of the State as well, to be present, that the largest number may share the benefits of the occasion. Then let every one, from this time forth, lay his plans with a view of being present.

### ANNUAL MEETING.

The thirteenth annual meeting of the Dental Society of the State of New York, will be held in the city of Albany, on the 11th and 12th of May, 1881, at 10 o'clock A. M.

The Sessions will be held in Geological Hall, on State Street. Quite a number of members will present interesting reports on assigned subject. Those having valuntary essays are requested to communicate with Dr. L. S. Straw, of Newburgh, at an early date. Visiting members of other Associations and States, will be especially welcome, and cordially received. The Delevan House will furnish accommodations to those attending the Society, at reduced rates.

New York is one of the few States having an efficient law regulating the practice of dentistry. The carrying out of its provisions is in the hands of the Society, and important work calls for attention at every meeting of this body. Through its Board of Censors it conferes the degree of "Master of Dental Surgery."

A large programme of interesting subjects will be presented for discussion.

### CONTENTS.

### COMMUNICATIONS.

A MONTHLY MAGAZINE DEVOTED TO THE INTERESTS OF

**THE DENTAL PROFESSION.**

*TERMS REDUCED TO $2.50 PER YEAR. SINGLE COPIES 25C.*

EDITED BY PROF. J. TAFT, D.D.S.

AND

*Published by SPENCER, CROCKER & CO., Ohio Dental Depot.*

The volume commences in January and closes in December.

The REGISTER being issued monthly is always fresh, therefore Indispensable to the practitioner who desires to keep posted up with the new inventions and improvements which are daily developed. Neither expense nor effort will be spared to give value and variety to its contents. Articles will be illustrated with well executed engravings whenever they will add to the interest or assist to explanation.

Its extensive circulation and frequency of issue make it one of the BEST DENTAL ADVERTISING MEDIUMS in the United States.

All subscriptions must begin with January or July.

Local or special notices, five lines or less, will be inserted for$3 each insertion; over five lines, 50 cts. per line.

All advertising bills payable in advance, or at furthest, after the issue of the first number containing the advertisement. All transient advertisements to be paid for in advance.

☞CONTRIBUTIONS SOLICITED.

Exclusively Editorial Communications should be addressed to Editor.

The latest number of the REGISTER may be ordered with or without oth-
er goods at any time, and all letters should be addressed to

*SPENCER, CROCKER & CO., Ohio Dental Depot, Cincinnati, O.*

